# Efficacy and safety of reduning injection for severe pneumonia: a systematic review and meta-analysis

**DOI:** 10.3389/fphar.2025.1591136

**Published:** 2025-08-08

**Authors:** Yan Wang, Baichuan Xu, Peng Zhang, Suyun Li, Yang Xie

**Affiliations:** ^1^ National Regional Traditional Chinese Medicine (Lung Disease) Diagnosis and Treatment Center, The First Affiliated Hospital of Henan University of Chinese Medicine, Zhengzhou, Henan, China; ^2^ The First Clinical College of Henan University of Traditional Chinese Medicine, Zhengzhou, Henan, China; ^3^ Collaborative Innovation Center for Chinese Medicine and Respiratory Diseases Co-Constructed by Henan Province & Education Ministry of P.R. Zhengzhou, Zhengzhou, Henan, China; ^4^ Henan Key Laboratory of Chinese Medicine for Respiratory Disease, Zhengzhou, Henan, China

**Keywords:** reduning injection, severe pneumonia, meta-analysis, systematic review, randomized controlled trial

## Abstract

**Objectives:**

Reduning (RDN) injection is a traditional Chinese medicine (TCM) extract injection commonly used as an adjunct therapy for severe pneumonia (SP) in clinical practice in China; however, further validation is required. This study aims to systematically assess the efficacy and safety of RDN injection in treating SP.

**Methods:**

The study was conducted using the data from CNKI, WanFang, VIP, SinoMed, PubMed, Embase, Cochrane Library, and Web of Science databases from their inception to 6 April 2024. We collected randomized controlled trials on the treatment of SP using RDN injection, and used Review Manager (version 5.3) for meta analysis. The primary outcomes was clinical efficacy. We used the Cochrane Risk of Bias Assessment Tool 2 (RoB 2) to assess the risk of bias for each study.

**Results:**

A total of 1,897 patients with SP from 22 studies were included. The meta-analysis results showed that the combination of RDN injection and conventional treatment or antibiotic treatment for patients with SP was superior to traditional and antibiotic treatments alone in terms of clinical efficacy [risk ratio = 1.25%; 95% confidence interval (CI) (1.20, 1.30); *p* < 0.00001], fever reduction time [mean difference (MD) = −1.55 days; 95% CI (−1.91, −1.18); *p* < 0.00001], cough disappearance time [MD = −1.97 days; 95% CI (−2.63, −1.31); *p* < 0.00001], lung rales disappearance time [MD = −2.39 days; 95% CI (−3.09, −1.70); *p* < 0.00001], chest X-ray improvement time [MD = −2.73 days; 95% CI (−2.96, −2.49); *p* < 0.00001], and hospitalization time [MD = −3.11 days; 95% CI (−3.80, −2.41); *p* < 0.00001]. Moreover, no significant improvement was observed in the Acute Physiology and Chronic Health Evaluation II (APACHE II) scale compared to the control group [MD = −2.45 points; 95% CI (−6.07, 1.17); *p* = 0.19]. Lastly, not all studies reported any serious adverse events; however, some studies did report adverse reactions.

**Conclusion:**

The administration of RDN injection as an adjunct therapy for SP can enhance clinical efficacy, reduce fever duration, accelerate cough resolution, shorten the time for lung rales to resolve, accelerate chest X-ray improvement, and decrease hospitalization duration. Further well-designed and standardized large sample clinical studies are needed for validation.

**Systematic review registration:**

PROSPERO, identifier CRD42024540365.

## 1 Introduction

Pneumonia is a common lung infection disease with infectivity, which disrupts the normal lives of patients (Liu et al., 2018). Pneumonia worsens and progresses to severe pneumonia (SP), causing obstruction of gas exchange in patients, leading to cerebral edema, hypovolemia, shock, and even other life-threatening conditions (Li et al., 2015). SP typically manifests systemically. Patients may experience high fever, severe chills, sweating, coughing up purulent or bloody sputum, fatigue, headache, muscle pain, and other symptoms (Ching and Pedersen, 2025). SP is a critical respiratory disease characterized by high mortality (Ramirez et al., 2011), multiple complications (Lee et al., 2016), a mortality rate of up to 30%–50% (Restrepo et al., 2010), and an increased burden on medical economics (Guan et al., 2025). Proactively investigating effective intervention strategies is crucial for reducing disease duration and mortality rates (Dinh et al., 2021).

The current guidelines indicate that SP is typically treated with antibiotics, mechanical ventilation, and corticosteroids. However, the use of glucocorticoids in SP is a conditional recommendation with low-quality evidence (Martin-Loeches et al., 2023), and prescribing excessive antibiotics may cause antibiotic resistance, potentially increasing mortality rates (Musher and Thorner, 2014; Aliberti et al., 2019). Studies have indicated that the use of corticosteroids to treat SP may have adverse reactions (Wu et al., 2023; See et al., 2024). Therefore, the prevention and treatment of SP remain a public health issue worthy of attention (Torres et al., 2019). The frequency of reports regarding the treatment of SP with Chinese herbal injections (CHIs) is rising, and their efficacy has been validated (Niu et al., 2022; Xiao et al., 2022). CHIs can proficiently address the medication challenges faced by patients who are comatose or have swallowing difficulties. Reduning (RDN) injection was approved as a traditional Chinese medicine in 2005 (Z20050217). RDN injection is effective in treating inflammatory diseases, with research indicating that Bcl-2, eNOS, PTGS2, PPARA, and MMPs play a crucial role in modulating the inflammatory processes associated with RDN injection compounds and metabolites (Xie et al., 2020). Recently, RDN injection has been widely used as an adjuvant therapy for SP in China, with confirmed efficacy of RDN injection in treating pneumonia (Cao et al., 2020). However, there is still a lack of systematic reviews on the use of RDN injection for SP. This study investigated randomized controlled trials (RCTs) of RDN injection as an adjunctive therapy for SP, systematically assessing its clinical efficacy and safety in treating SP, and providing guidance for the clinical management of SP.

## 2 Methods

This study was conducted in accordance with the Preferred Reporting Items for Systematic Reviews and Meta-Analyses (PRISMA 2020) guidelines (Page et al., 2021), and the protocol has been registered in the International Prospective Register of Systematic Reviews (PROSPERO) (CRD42024540365).

### 2.1 Eligibility criteria

The inclusion criteria were: (1) All studies must be RCTs without language restrictions; (2) participants must be diagnosed with SP according to any internationally accepted guidelines, aged over 18 years, irrespective of gender or ethnicity; and (3) the experimental group received either combined or non-combined conventional treatment with RDN Injection, while the control group received either conventional treatment or a placebo.

### 2.2 Exclusion criteria

The exclusion criteria were: (1) Duplicate publications; (2) Integration of literature on other diseases; (3) Animal experimental studies; and (4) Inaccessibility of original literature.

### 2.3 Search strategy

We searched across the China National Knowledge Infrastructure (CNKI), WanFang, the Chinese Scientific Journal Database (VIP), SinoMed, PubMed, Embase, the Cochrane Library, and Web of Science databases, with no language restrictions, from the inception of the databases until 6 April 2024. The search terms were: “Reduning” or “Reduning injection” and “Severe pneumonia” or “Serious pneumonia” and “random” or “randomized controlled trial” or “controlled trial” (Supplementary Table S1).

### 2.4 Research selection

Two researchers (YW and BCX) independently searched, screened, and extracted data according to the inclusion and exclusion criteria. Initially, repetitive and unrelated literature was excluded based on the title and abstract; pertinent literature, along with its references, was then investigated. The full text of qualified literature was read individually. If a disagreement arose between the two researchers, a third researcher (PZ) was consulted to reach a consensus.

### 2.5 Data extraction

The two researchers independently extracted data, including the lead author, publication date, country, research type, research duration, diagnostic criteria, sample size, age, sex, outcome indicators, and additional information, using a pre-designed form according to the PRISMA 2020 guidelines (Page et al., 2021), and cross-checked them. When two researchers encountered disagreements, a third researcher (PZ) was tasked with reaching a consensus.

### 2.6 Outcomes

The primary outcome measure was clinical efficacy. The secondary outcome measures were duration until defervescence, to cough resolution, time to disappearance of lung rales, time to improvement in chest X-ray, length of hospitalization, Acute Physiology and Chronic Health Evaluation II (APACHE II) score, and adverse reactions.

### 2.7 Risk-of-bias assessment

This study employed the Cochrane Risk of Bias Assessment Tool 2 (RoB 2) (Sterne et al., 2019) to evaluate the included literature, focusing on the randomization process, deviations from intended interventions, missing outcome data, outcome measurement, and selection of reported results.

### 2.8 Statistical analysis

A meta-analysis was conducted using Review Manager (version 5.3). Risk ratio was used for ranked variables, and MD was used for continuous variables. This study utilized 95% CI to evaluate all outcome measures. *I* ^2^ < 50% indicated low heterogeneity, and thus, a fixed-effects model was used; *I* ^2^ ≥ 50% indicated high heterogeneity, and a random-effects model was used. When *I* ^2^ was non-zero and the meta-analysis encompassed four or more studies, a subgroup analysis was performed based on RDN dosage or treatment duration. A sensitivity analysis was performed to sequentially exclude individual studies. A funnel plot was used to analyze for any publication bias. The Grading of Recommendations, Assessment, Development, and Evaluation (GRADE) (Gonzalez-Padilla and Dahm, 2021) was used to evaluate the quality of outcome measures. The quality of evidence was categorized into four levels: high, moderate, low, and very low.

## 3 Results

### 3.1 Search results

**FIGURE 1:**
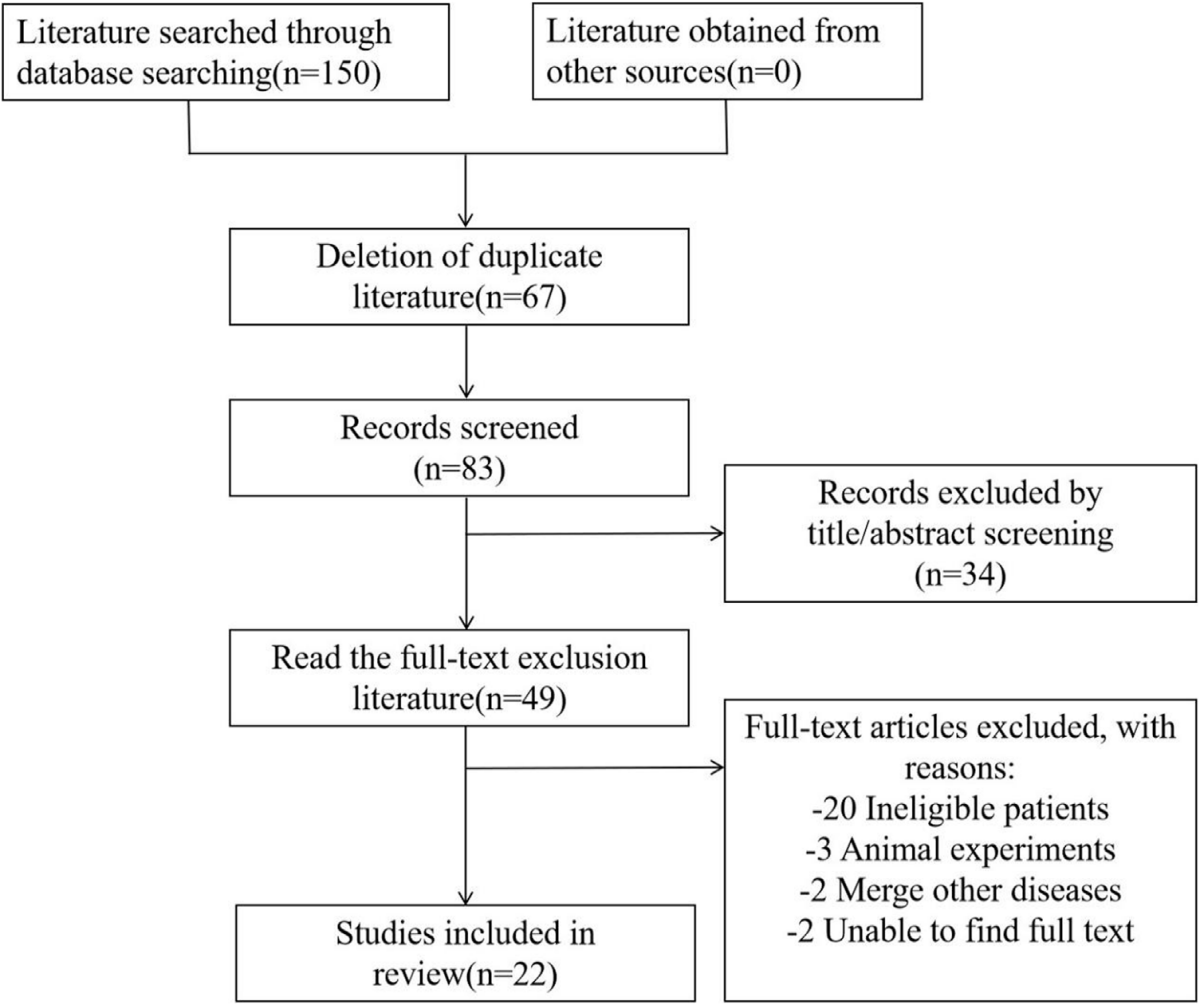
Literature selection process.

A total of 150 articles were retrieved, and 67 duplicate articles were excluded. Thirty-four irrelevant articles were excluded after reading the titles and abstracts. After analyzing the full text, 27 articles were excluded, including 20 studies focused on children, 3 articles on animal experiments, 2 articles that integrated on other diseases, and 2 articles for which the original text was inaccessible. Finally, we included 22 articles. The literature screening process is shown in Figure 1.

### 3.2 Characteristics of studies

A total of 22 studies RCTs were included (Sun et al., 2012; Yin et al., 2013; Liu and Liu, 2014; Li, 2014; Lin, 2015; Zhang et al., 2016; Nie, 2017; Zhou, 2017; Ding, 2017; Fang et al., 2018; Chen, 2018; Zhu et al., 2019; Tan et al., 2019; Yan et al., 2019; Shan, 2020; Wang et al., 2020; Bai and Xu, 2021; Pan, 2022; Guo and Huo, 2022; Li and Shao, 2022; Xing and Lin, 2022; Wang SG. et al., 2023). All studies were conducted in China, encompassing 1897 patients with SP, with 950 in the experimental group and 947 in the control group. The minimum sample size was 60 and the maximum sample size was 140. The control group received treatment methods that include conventional treatment, antibiotics, a combination of conventional treatment and antibiotics, a combination of conventional treatment and protease inhibitors, and a combination of conventional treatment and bronchoalveolar lavage. The treatment methods received by the experimental group include RDN injection combined with conventional treatment, RDN injection with antibiotics, RDN injection with conventional treatment and antibiotics, RDN injection with conventional treatment and immunosuppressants, RDN injection with conventional treatment and a protease inhibitor, and RDN injection combined with conventional treatment and bronchoalveolar lavage. All patients receiving treatment with RDN injection received an intravenous injection of RDN once daily, with injection doses of 0.6 mL/kg, 20 mL, and 30 mL. The duration of treatment ranged from 3 to 14 days. The treatment duration for 13 studies exceeded 7 days, while the treatment duration for 9 studies was 7 days or less. The basic characteristics included in the study are shown in Table 1.

**TABLE 1:** Basic characteristics of included studies.

Study	Sample (E/C)	Gender (M/F)	Age (years)	Intervention	Study period	Outcomes
Sun et al. (2012)	33/31	33/31	–	E: RDN+CT	7–10	①⑦⑧
				C: CT		
Yin et al. (2013)	40/40	E: 25/15	E: 45.6±8.1	E: RDN+CT	10	①②③④⑤
		C: 30/10	C: 50.6±6.9	C: CT		
Liu and Liu (2014)	34/36	E: 20/14	E: 35.42±1.66	E: RDN+CT+AB	12	①②④⑤⑥
		C: 18/18	C: 36.32±1.56	C: CT+AB		
Li (2014)	68/72	E: 40/28	E: 34.22±1.77	E: RDN+CT+AB	12	①②④⑤⑥
		C: 36/36	C: 35.22±1.66	C: CT+AB		
Lin (2015)	36/30	–	–	E: RDN+CT+Ig	5	②③④⑤⑧
				C: CT+Ig		
Zhang et al. (2016)	43/43	E: 25/18	E: 63.29±9.03	E: RDN+CT	7–10	①⑧
		C: 23/20	C: 62.86±8.85	C: CT		
Nie (2017)	42/42	E: 22/20	E: 51.33±5.73	E: RDN+CT	3	①⑧
		C: 24/18	C: 52.10±5.68	C: CT		
Zhou (2017)	39/39	E: 20/19	E: 73.54±12.09	E: RDN+CT	14	①②⑤
		C: 22/17	C: 72.68±11.26	C: CT		
Ding (2017)	50/50	E: 29/21	E: 71.37±5.71	E: RDN+CT	10	①②③④⑤
		C: 31/19	C: 71.54±5.69	C: CT		
Fang et al. (2018)	36/36	E: 21/15	E: 34.1±3.2	E: RDN+CT+AB	12	①②④⑤⑥⑧
		C: 22/14	C: 33.8±2.7	C: CT+AB		
Chen (2018)	48/48	E: 29/19	–	E: RDN+CT+PI	7	①⑧
		C: 30/18		C: CT		
Zhu et al. (2019)	43/43	E: 24/19	E: 37.82±6.09	RDN+CT	14	①②③④⑥
		C: 23/20	C: 37.51±6.23	C: CT		
Tan et al. (2019)	52/51	E: 28/24	E: 63.52±7.01	E: RDN+CT+BAL	7	①⑦⑧
		C: 28/23	C: 64.01±6.87	C: CT+BAL		
Yan et al. (2019)	42/42	E: 28/14	E: 56.10±8.23	E: RDN+AB	7	①②③④⑥
		C: 24/18	C: 56.02±8.37	C: AB		
Shan (2020)	50/50	E: 29/21	E: 57.5±5.8	E: RDN+AB	7	①②③④
		C: 28/22	C: 57.9±6.1	C: AB		
Wang et al. (2020)	30/30	E: 16/14	E: 59.97±4.03	E: RDN+CT	7	①⑦
		C: 15/15	C: 61.03±4.07	C: CT		
Bai and Xu (2021)	32/32	–	E: 55.17±7.55	E: RDN+AB	7	①
			C: 55.28±7.49	C: AB		
Pan (2022)	40/40	E: 26/14	E: 56.29±4.34	E: RDN+CT+AB	12	①②④⑤⑥
		C: 27/13	C: 57.13±3.68	C: CT+AB		
Guo and Huo (2022)	57/57	E: 34/23	E: 34.42±1.98	E: RDN+CT+AB	12	①②④⑤⑥⑧
		C: –	C: 33.85±2.05	C: CT+AB		
Li and Shao (2022)	43/43	E: 28/15	E: 37.82±6.90	E: RDN+AB	14	①②③④⑤⑥⑦⑧
		C: 26/17	C: 37.16±6.51	C: AB		
Xing and Lin (2022)	45/45	E: 27/18	E: 57.93±3.54	E: RDN+AB	5	②③④⑧
		C: 26/19	C: 57.89±3.57	C: AB		
Wang et al. (2023)	47/47	E: 30/17	E: 53.20±5.68	E: RDN+AB	10	①②③④⑧
		C: 28/19	C: 53.04±5.42	C: AB		

Abbreviations: E, experimental group; C, control group; AB, antibiotics; CT, conventional therapy; Ig, immunoglobulin; PI, protease inhibitor; BAL, bronchoalveolar lavage; RDN, Reduning. ① Clinical efficacy; ② Time for defervescence; ③ Time for disappearance of cough; ④ Time for disappearance of lung rales; ⑤ Improvement time of chest X-ray; ⑥ The hospitalization time; ⑦ Acute Physiology and Chronic Health Evaluation (APACHE Ⅱ); ⑧Adverse reactions.

### 3.3 Risk-of-bias assessment

**FIGURE 2:**
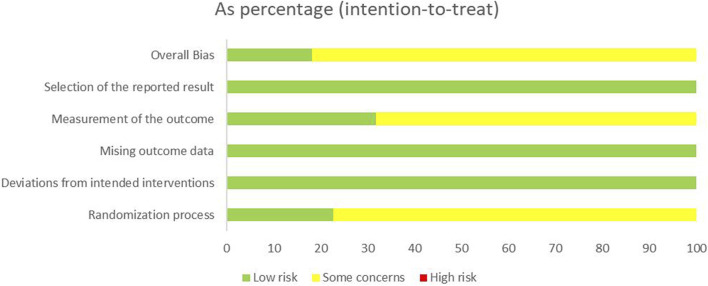
Risk of bias summary of included studies.

**FIGURE 3:**
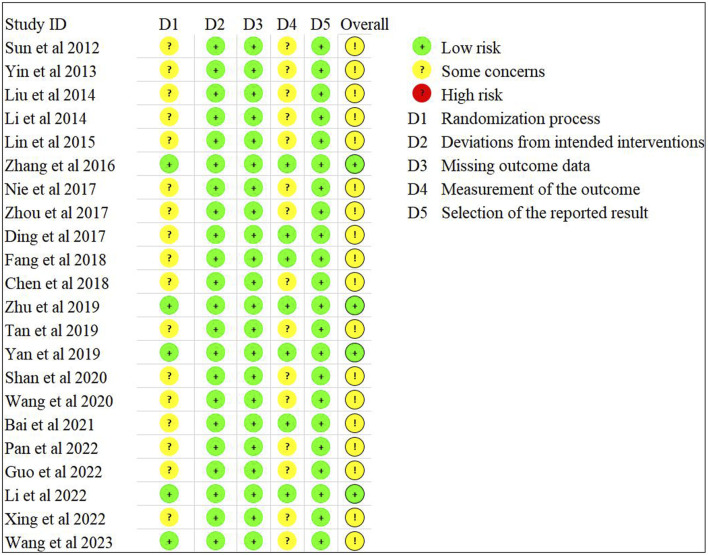
Risk of bias in included studies.

Sixteen studies (Sun et al., 2012; Yin et al., 2013; Zhang et al., 2016; Nie, 2017; Zhou, 2017; Ding, 2017; Fang et al., 2018; Chen, 2018; Zhu et al., 2019; Yan et al., 2019; Shan, 2020; Wang et al., 2020; Bai and Xu, 2021; Pan, 2022; Li and Shao, 2022; Wang SG. et al., 2023) employed a random grouping method, of which only seven studies (Zhang et al., 2016; Zhou, 2017; Chen, 2018; Zhu et al., 2019; Yan et al., 2019; Li and Shao, 2022; Wang SG. et al., 2023) utilized a random number table method, while nine studies (Sun et al., 2012; Yin et al., 2013; Nie, 2017; Ding, 2017; Fang et al., 2018; Shan, 2020; Wang et al., 2020; Bai and Xu, 2021; Pan, 2022) only mentioned “random” without detailing the specific randomization method. The 22 studies neither specified whether allocation concealment and blinding were implemented nor presented evidence of selective reporting or other possible sources of bias. The risk bias assessment is illustrated in Figures 2, 3.

### 3.4 Clinical efficacy

**FIGURE 4:**
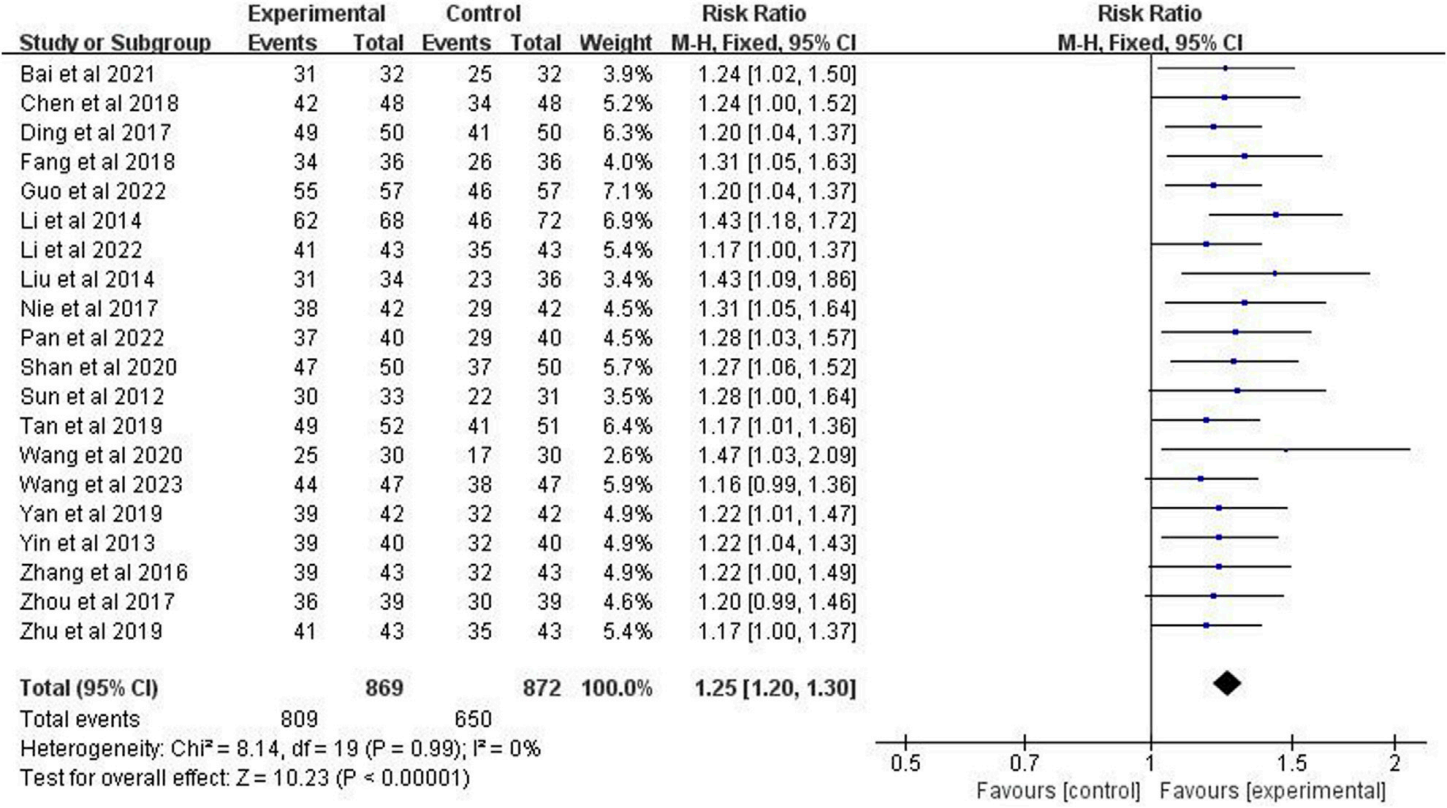
Forest plot of clinical efficacy.

Twenty studies (*n* = 1,741) were included to report clinical efficacy. The meta-analysis revealed *p* = 0.99 and *I* ^2^ = 0%, indicating low heterogeneity and the application of a fixed-effects model. It also indicated that the clinical efficacy rate of the experimental group is significantly higher than the control group (risk ratio = 1.25%; 95% CI [1.20, 1.30]; *p* < 0.00001) (Figure 4).

### 3.5 Time for defervescence

**FIGURE 5:**
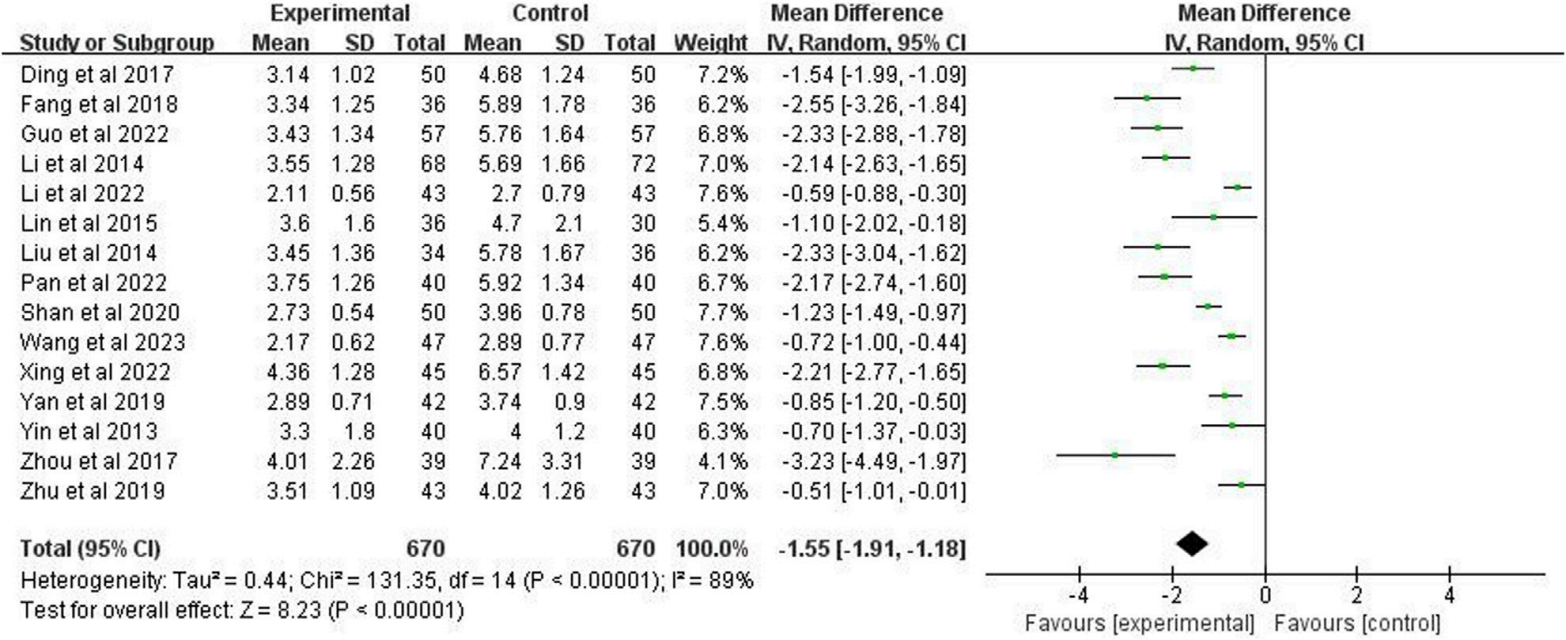
Forest plot of time for defervescence.

Fifteen studies (*n* = 1,340) were included to report the time to defervescence. The analysis demonstrated a *p* < 0.00001 and *I* ^2^ = 89%, indicating high heterogeneity, using a random-effects model. It further indicated that the fever reduction time in the experimental group was significantly shorter than the control group (MD = −1.55 days; 95% CI [−1.91, −1.18]; *p* < 0.00001) (Figure 5).

### 3.6 Time for cough disappearance

**FIGURE 6:**
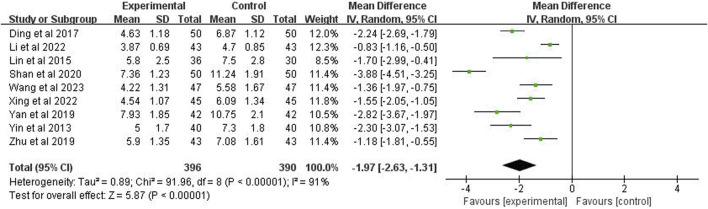
Forest plot of time for disappearance of cough.

Nine studies (*n* = 786) reported the time for the disappearance of cough. The meta-analysis showed, using a random effects model, *p* < 0.00001 and *I* ^2^ = 91%, indicating high heterogeneity. The analysis also demonstrated that the cough disappearance time in the experimental group was significantly shorter than the control group (MD = −1.97 days; 95% CI [−2.63, −1.31]; *p* < 0.00001) (Figure 6).

### 3.7 Time for disappearance of lung rales

**FIGURE 7:**
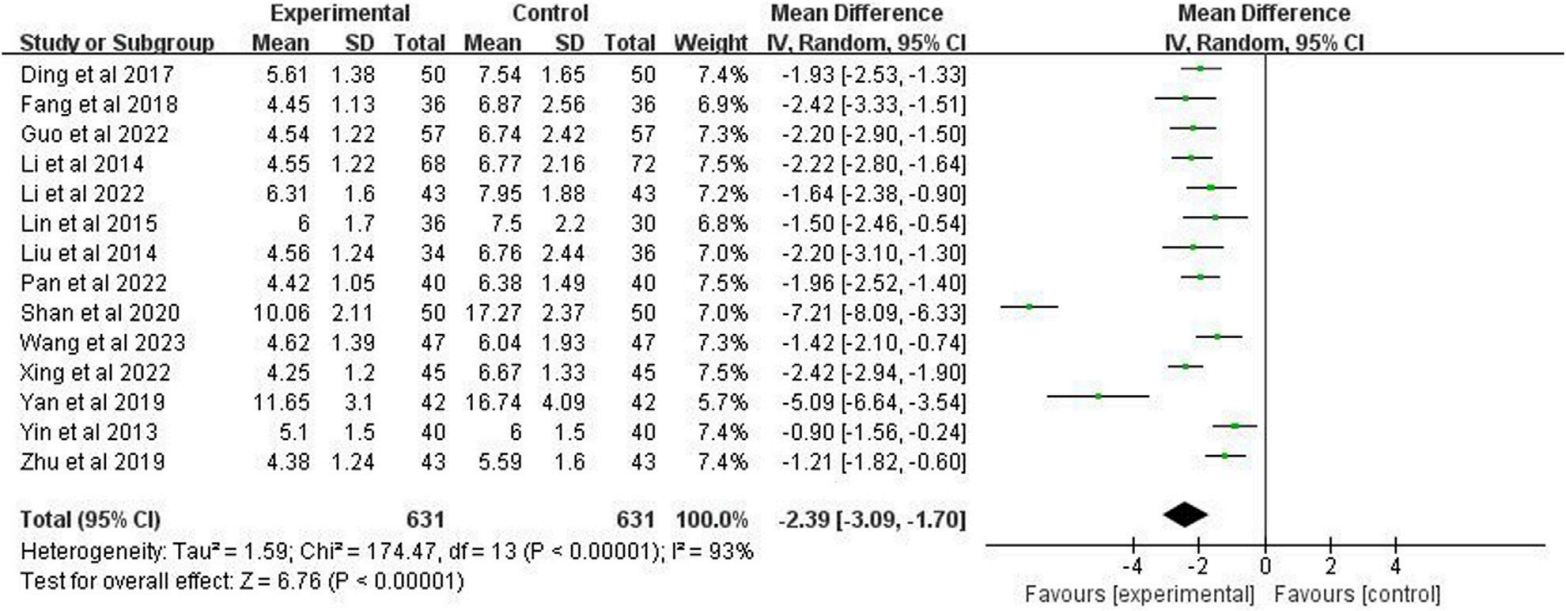
Forest plot of time for disappearance of lung rales.

The time to disappearance of lung rales was included in 14 studies (*n* = 1,262). Through a random effects model, the meta-analysis demonstrated a *p* < 0.00001 and *I* ^2^ = 93%, indicating high heterogeneity. The analysis further showed that the disappearance time of lung rales in the experimental group was significantly shorter than the control group (MD = −2.39 days; 95% CI [−3.09, −1.70]; *p* < 0.00001) (Figure 7).

### 3.8 Time for improvement time of chest X-ray

**FIGURE 8:**
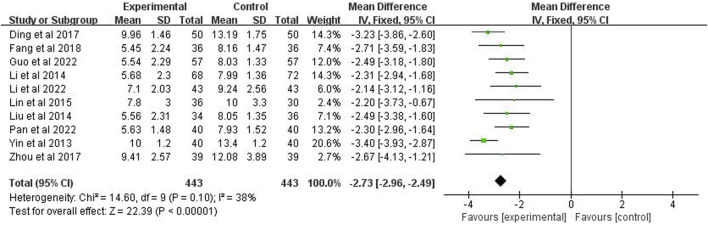
Forest plot of improvement time of chest X-ray.

The improvement time of chest X-ray was reported in studies (*n* = 886). The analysis showed *p* = 0.10 and *I* ^2^ = 38% after applying a fixed effects model, indicating low heterogeneity. It also showed that the improvement time of chest X-rays in the experimental group was significantly shorter than the control group (MD = −2.73 days; 95% CI [−2.96, −2.49]; *p* < 0.00001) (Figure 8).

### 3.9 The hospitalization time

**FIGURE 9:**
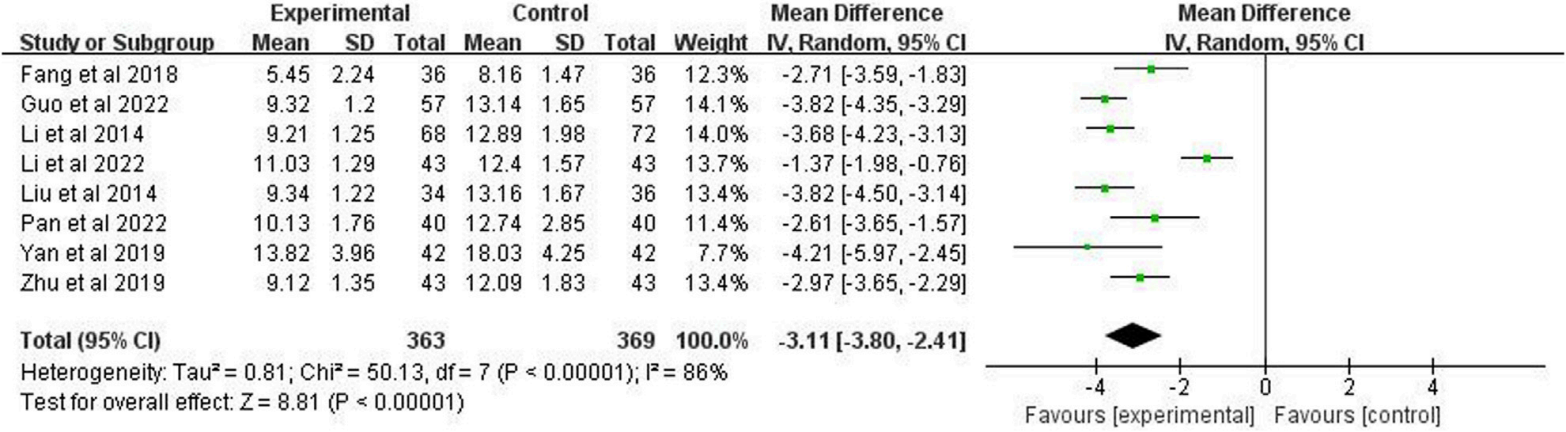
Forest plot of the hospitalization time.

Eight studies (*n* = 732) reported the hospitalization time. After using the random effects model, the meta-analysis showed *p* < 0.00001 and *I* ^2^ = 86%, indicating high heterogeneity. It also showed that the hospitalization time of the experimental group was significantly shorter than the control group (MD = −3.11 days; 95% CI [−3.80, −2.41]; *p* < 0.00001) (Figure 9).

### 3.10 APACHE Ⅱ

**FIGURE 10:**
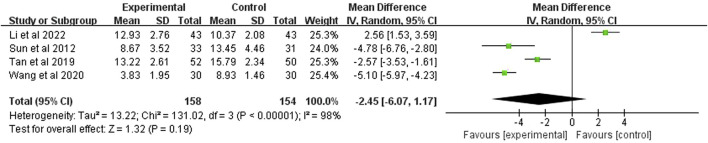
Forest plot of APACHEⅡ.

APACHE II score was reported in 4 studies (*n* = 312). The analysis, after using a random effects model, showed *p* < 0.00001 and *I* ^2^ = 98%, indicating high heterogeneity. It also demonstrated that the APACHE II score of the experimental group was significantly higher than the control group (MD = −2.45 points; 95% CI [−6.07, 1.17]; *p* = 0.19) (Figure 10).

### 3.11 Adverse reactions

Adverse reactions were documented in 13 studies, with 2 studies indicating no adverse reactions, while 11 studies (Sun et al., 2012; Lin, 2015; Zhang et al., 2016; Nie, 2017; Zhou, 2017; Fang et al., 2018; Chen, 2018; Tan et al., 2019; Yan et al., 2019; Guo and Huo, 2022; Li and Shao, 2022; Xing and Lin, 2022; Wang SG. et al., 2023) reported reactions such as nausea, diarrhea, rash, headache, and dizziness. The remaining nine studies also reported no adverse reactions. The incidence of adverse events in the experimental group was 8.88% (50/563), whereas in the control group it was 9.21% (51/554) (Table 2). Additional evidence is necessary to establish the safety of RDN injection in the treatment of SP.

**TABLE 2:** Occurrence of adverse reactions.

Adverse reactions	Incidence rate (n, %)	
	Experimental	Control
Dry mouth	5 (0.89)	5 (0.90)
Nausea	11 (1.95)	8 (1.44)
Diarrhea	7 (1.24)	7 (1.26)
Dizziness	2 (0.36)	1 (0.18)
Skin itching	3 (0.53)	0
Increased heart rate	1 (0.18)	1 (0.18)
Edema	2 (0.36)	4 (0.72)
Rash	3 (0.53)	8 (1.44)
Palpitations	2 (0.36)	0
Chest tightness	1 (0.18)	0
Constipation	1 (0.18)	2 (0.36)
Indigestion	1 (0.18)	1 (0.18)
Headache	3 (0.53)	5 (0.90)
Thrombophlebitis	1 (0.18)	2 (0.36)
Liver and kidney injury	1 (0.18)	1 (0.18)
Muscle spasm	1 (0.18)	1 (0.18)
Gastrointestinal reactions	3 (0.53)	1 (0.18)
Dyspnea	1 (0.18)	2 (0.36)
Gastric distension and nausea	0	1 (0.18)
Vomiting	0	1 (0.18)
Elevated alanine aminotransferase	1 (0.18)	0

### 3.12 Subgroup analyses

The subgroup analysis indicated that the duration of improvement in chest X-ray following treatment of SP with 30 mL of RDN Injection was superior to that observed with 20 mL and 0.6 mL/kg (MD −3.33 vs. −2.41 vs. −2.20, interaction *p* = 0.001). The analysis results for other subgroups showed that the p-value for the interaction was greater than 0.05, indicating no subgroup differences (Table 3).

**TABLE 3:** Results of subgroup analysis.

Outcomes	Subgroup	Studies	Total	ES	Heterogeneity	Interaction p value
		*n*		(95% CI)	*I* ^2^	
Time for defervescence						
The dosage	20 mL	11	1004	MD −1.59 (−2.04, −1.15)	91%	0.61
	30 mL	2	180	MD −1.16 (−1.98, −0.34)	76%	
	0.6 mL/kg	2	156	MD −1.72 (−2.80, −0.64)	76%	
Treatment duration	>7 days	11	1000	MD −1.64 (−2.14, −1.14)	91%	0.41
	≤7 days	4	340	MD −1.33 (−1.86, −0.81)	82%	
Time for disappearance of cough						
The dosage	20 mL	5	450	MD−1.99 (−3.14, −0.85)	95%	0.09
	30 mL	2	180	MD −2.26 (−2.64, −1.87)	0%	
	0.6 mL/kg	2	156	MD −1.57 (−2.04, −1.10)	0%	
Treatment duration	>7 days	5	446	MD −1.56 (−2.21, −0.91)	87%	0.18
	≤7 days	4	340	MD −2.51 (−3.77, −1.26)	91%	
Time for disappearance of lung rales						
The dosage	20 mL	10	926	MD −2.70 (−3.67, −1.73)	94%	0.20
	30 mL	2	180	MD −1.42 (−2.43, −0.42)	81%	
	0.6 mL/kg	2	156	MD −2.05 (−2.94, −1.17)	63%	
Treatment duration	>7 days	10	922	MD −1.78 (−2.09, −1.48)	51%	0.09
	≤7 days	4	340	MD −4.04 (−6.65, −1.43)	97%	
Improvement time of chest X-ray						
The dosage	20 mL	7	640	MD −2.41 (−2.71, −2.11)	0%	0.001
	30 mL	2	180	MD −3.33 (−3.73, −2.93)	0%	
	0.6 mL/kg	1	66	MD −2.20 (−3.73, −0.67)	-	
Treatment duration	>7 days	9	820	MD −2.74 (−2.98, −2.50)	43%	0.50
	≤7 days	1	66	MD −2.20 (−3.73, −0.67)	-	
The hospitalization time						
Treatment duration	>7 days	7	648	MD −3.01 (−3.74, −2.29)	88%	0.22
	≤7 days	1	84	MD −4.21 (−5.97, −2.45)	-	
APACHE Ⅱ						
Treatment duration	>7 days	2	150	MD −1.06 (−8.25, 6.13)	98%	0.47
	≤7 days	2	162	MD −3.84 (−6.32, −1.36)	93%	

Abbreviations: ES, effect estimate; MD, mean difference; CI, confidence interval.

### 3.13 Sensitivity analysis

An individual exclusion method was employed for the sensitivity analysis, and the results revealed no significant changes, indicating the stability of the findings.

### 3.14 Publication bias

**FIGURE 11:**
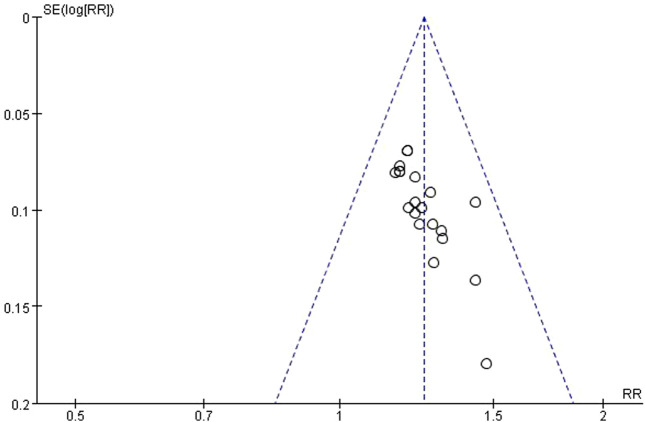
Funnel plot of clinical efficacy.

A plotted a funnel plot of the main indicators of clinical efficacy was generated, and the results showed poor symmetry, with the studies distributed on both sides of the vertical line. Additionally, Begg and Egger tests were conducted, revealing a p-value of 0, signifying publication bias potentially associated with the subpar quality of the literature (Figure 11).

### 3.15 Quality of evidence

The GRADE evaluation indicated that the evidence for clinical efficacy and the time to improvement in chest X-ray quality was classified as moderate. In contrast, other outcome measures were deemed to be of very low quality (Table 4).

**TABLE 4:** GRADE evidence profile.

Certainty assessment							No of patient		Effect estimate		Certainty	Importance
No of studies	Study design	Risk of bias	Inconsistency	Indirectness	Imprecision	Other considerations	E	C	Relative (95% CI)	Absolute (95% CI)		
Clinical efficacy												
20	RCT	Serious	Not serious	Not serious	Not serious	None	809/869 (93.1%)	650/872 (74.5%)	RR 1.25 (1.20, 1.30)	186 per 1,000 (149, 224)	Moderate	CRITICAL
Time for defervescence												
15	RCT	Serious^a^	Very serious^b^	Not serious	Not serious	None	670	670	-	MD −1.55 (−1.91, −1.18)	Very low	IMPORTANT
Time for disappearance of cough												
9	RCT	Serious^a^	Very serious^b^	Not serious	Not serious	None	396	390	-	MD −1.97 (−2.63, −1.31)	Very low	IMPORTANT
Time for disappearance of lung rales												
14	RCT	Serious^a^	Very serious^b^	Not serious	Not serious	None	631	631	-	MD −2.39 (−3.09, −1.70)	Very low	IMPORTANT
Improvement time of chest X-ray												
10	RCT	Serious^a^	Not serious	Not serious	Not serious	None	443	443	-	MD −2.73 (−2.96, −2.49)	Moderate	IMPORTANT
The hospitalization time												
8	RCT	Serious^a^	Very serious^b^	Not serious	Not serious	None	363	369	-	MD −3.11 (−3.80, −2.41)	Very low	IMPORTANT
APACHEⅡ												
4	RCT	Serious^a^	Very serious^b^	Not serious	Serious^c^	None	158	154	-	MD −2.45 (−6.07, 1.17)	Very low	IMPORTANT

Abbreviations: CI, confidence interval; MD, mean difference; RR, risk ratio; E, Experimental; C, Control. Explanations: a. The results of the COCHRANE, bias risk assessment show that most (2/3) of the information comes from moderate bias; b. Heterogeneity test shows a high I^2^ value, with the forest plot indicating I^2^ > 75%; c. 95% confidence interval CI, over equivalent line.

## 4 Discussion

SP is a significant respiratory ailment characterized by complex and challenging etiologies, affecting people of all ages globally. Patients with SP admitted to the ICU are associated with high mortality rates (Espinoza et al., 2019). Antibiotic treatment is the foundation for treating SP, should be initiated as early as possible (Garnacho-Montero et al., 2018). Due the long-term use of antibiotics, the medical costs associated with treating SP are relatively high (Welte, 2016). CHIs have certain advantages during the treatment of SP. Guidelines (Wang M. et al., 2023) advocate for the administration of Tanreqing, RDN, Xuebijing, Shenfu, and Shenmai injections for the treatment of SP in adults. In recent years, CHIs have been widely used in clinical practice (Deng and Chen, 2021; Jiang et al., 2024; Geng, 2024), with the literature continually updated. Numerous studies have shown that RDN injection can treat respiratory system diseases (Ma et al., 2021; Wang Z. et al., 2023; Wang et al., 2022). Despite clinical studies on the treatment of SP with RDN Injection, its efficacy and safety remain ambiguous. Therefore, this study sought to identify RCTs regarding the treatment of SP with RDN injection, aiming to guide the clinical management of SP.

The pathogen of SP can induce a high inflammatory response. RDN injection can address SP for the following reasons: The primary constituents of RDN Injection include *Gardenia jasminoides*, *Lonicera japonica*, and *Artemisia annua*, all of which possess anti-inflammatory, antibacterial, antiviral, and additional properties (Xu et al., 2009). Studies have shown that their active ingredients mainly include iridoids, lignans, coumarins, sesquiterpenes, flavonoids, caffeoylquinic acid and phenolic acids (Liu et al., 2015). The main iridoid in *Gardenia* and chlorogenic acid in *Lonicera japonica* can inhibit the macrophage response associated with inflammatory diseases, reduce the release of inflammatory factors, and inhibit infections (Cao et al., 2021). A study investigated the antipyretic and anti-inflammatory properties of RDN injection by screening its active ingredients using a mouse endotoxin shock model, resulting in the identification of two novel terpenoid compounds: designated geniposide A and identified as (1*R*,7*R*,8*S*,10*R*)-7,8,11-trihydroxy-4-guaiacin-3-one (Li et al., 2020). Research has demonstrated that RDN effectively regulates the metabolic disorders induced by endogenous components in febrile rats treated with dry yeast (Gao et al., 2020). Its antipyretic effect is mainly related to the regulation of amino acids, lipids, and energy metabolism. Network pharmacology studies have shown that RDN can regulate various biological processes and treat inflammation at the systemic level (Xie et al., 2020). These mechanisms confirm the authenticity of treating SP with RDN injection. Currently, there is a gradual increase in the focus on RDN injection.

RCTs were searched for the treatment of SP with RDN injection, and data from 1,897 patients with SP were studied. Meta-analysis results showed that RDN injection can improve clinical efficacy, reduce fever duration, shorten cough resolution time, decrease lung rales resolution time, enhance chest X-ray improvement, and decrease hospitalization time in patients with SP. There was no statistically significant difference between the two groups in terms of improving APACHE II scores, which may be related to the small number of included studies and their relatively small sample sizes. We recommend adjusting the dosage of RDN injection according to the patient’s condition, generally 20–30 mL, continuously used for 7–14 days. Although this meta-analysis provides valuable insights into the efficacy and safety of RDN injection in clinical practice, the included studies were conducted solely in China, which may limit the external validity of our results. Cultural and genetic differences may influence the safety and efficacy of RDN injection. Future research should conduct trials in different populations to evaluate the effectiveness and safety of RDN injection for various ethnic and cultural backgrounds.

Regarding safety, although the adverse reactions are mild and self-resolving, this study cannot conclude that the use of RDN injection does not increase patient adverse reactions. Though no serious adverse events were reported in the included studies, the lack of detailed adverse event reporting in some studies is a notable limitation, which can lead to an underestimation of the true incidence of adverse events associated with RDN injection. We recommend that future trials on RDN injection adopt standardized protocols for collecting and reporting adverse events. Recommendations entail the implementation of standardized adverse event forms, precise definitions of adverse events, and uniform follow-up procedures to monitor and document such occurrences.

This research suggests that certain studies provide incomplete descriptions of randomization and blinding methods, which may lead to multifaceted biases that affect the results of this study. For instance, if randomization was not properly conducted, there might be systematic differences between the intervention and control groups at baseline, which could affect the observed treatment effects. Similarly, the absence of blinding may result in biased evaluations of outcomes, either by altering patient behavior or compromising the precision of outcome measurements. Inadequate allocation concealment may lead to outcome assessors being biased in their detection or reporting of results if they are aware of the treatment allocation (Jadad et al., 1996), which can result in overestimation or underestimation of the true therapeutic effect. Future research should aim to address these issues to provide more reliable evidence.

We have examined differences in patient characteristics, such as disease severity and comorbidities, across the included investigations. However, due to insufficient information, it is not possible to ascertain whether these are heterogeneous sources. Differences in antibiotic use may lead to high heterogeneity. There were discrepancies in the diagnostic criteria, which may contribute to heterogeneity in the results. Despite the efforts to investigate their potential sources, some unexplained heterogeneity remains in this meta-analysis. The high heterogeneity suggests that the effectiveness of interventions may vary among different populations or environments. This indicates that the findings from this study should be applied with caution in clinical practice. Clinicians must evaluate the distinct attributes of their patient demographic and the contextual factors of their practice when determining the appropriateness of the intervention. Future research should aim to identify and address the sources of heterogeneity to provide more reliable and generalizable evidence. Moreover, a substantial publication bias (*p* = 0) was identified in the analysis of the clinical efficacy of the primary indicator, suggesting that studies yielding negative or inconclusive results are unlikely to be disseminated. Therefore, there is a potential risk of overestimating the positive effects of treatment. Hence, more comprehensive and rigorous research is needed in the future to address this issue.

The quality of evidence of several studies included in this article is low. Biases and methodological limitations in the primary studies may result in an inaccurate estimation of the actual treatment effects. Clinicians should consider the limitations of evidence when making treatment decisions. They may need to rely on additional sources of information, such as expert opinion and clinical experience, to guide their practice. It is also important to monitor the emerging evidence from higher-quality studies to inform future clinical guidelines. It is recommended that future RCTs include the following features: strict randomization techniques and blinding of participants and outcome assessors to treatment allocation. This will help minimize the risk of selection and performance bias. Future trials should detect clinically significant differences, which requires careful planning and calculation of sample size based on expected effect size and variability. Consistent and standardized outcome measures should be used in all studies to facilitate the comparison and aggregation of results, including the use of validated tools to evaluate primary and secondary outcomes. Detailed adverse event reports and long-term follow-up should also be provided. By combining these features, future RCTs will provide better quality evidence, offering more reliable information for clinical practice and future research.

## 5 Limitations

This study has certain limitations: (1) The number of included studies is limited, the sample size is diminutive, and the overall quality of the literature is substandard; (2) some studies had incomplete descriptions of randomization methods; (3) all studies did not mention blinding, which may result in some bias in the results; (4) the heterogeneity cannot be determined through subgroup analysis; and (5) the included studies did not mention any long-term efficacy indicators, such as survival rate and mortality rate, and the treatment time was relatively short, making long term efficacy unclear.

## 6 Conclusion

In conclusion, the meta-analysis results indicate that the use of RDN injection as an adjuvant therapy for SP can enhance clinical efficacy, reduce fever duration, shorten cough resolution time, decrease lung rale resolution time, improve chest X-ray results, and decrease hospitalization time. Future validation necessitates the execution of more meticulously designed and standardized large-scale clinical studies.
