# Supplementary material for: Efficacy and safety of reduning injection for severe pneumonia: a systematic review and meta-analysis
Source: Front Pharmacol. 2025 Aug 8;16:1591136. doi: 10.3389/fphar.2025.1591136 (PMC12371217; doi:10.3389/fphar.2025.1591136)
Supplement: Supplementary file 1 [file DataSheet1.docx]

**Supplementary materials**

| **Section** | **Title** | **Page** |
| --- | --- | --- |
| 1 | Full search strategy in each database | 1 |
| 2 | PRISMA 2020 Checklist | 2-4 |
| 3 | Reduning injection | 5-8 |

1. Full search strategy in each database

Table S1 Full search strategy in each database

| **Database** | **Search strategy** |
| --- | --- |
| **PubMed** | (((Reduning[Title/Abstract]) OR (Reduning injection[Title/Abstract])) AND((Severe pneumonia[Title/Abstract]) OR (Serious pneumonia[Title/Abstract) AND(((Random[Title/Abstract]) OR (Randomized controlled trial[Title/Abstract])) OR (Controlledtrial[Title/Abstract])) |
| **Embase** | (Reduning.ab.ti. OR Reduning injection.ab.ti.) AND (Severe pneumonia.ab.ti. OR Serious pneumonia.ab.ti.) |
| **The Cochrane Library** | #1 Reduning OR Reduning injection  #2 Severe pneumonia OR Serious pneumonia  #3 Random OR Random controlled trial OR Controlled trial  #4 #1 AND #2 AND #3 |
| **Web of science** | #1 Reduning OR Reduning injection  #2 Severe pneumonia OR Serious pneumonia  #3 #1 AND #2 |
| **SinoMed** | (热毒宁[全部字段:智能] OR 热毒宁注射液[全部字段:智能] OR 热毒宁注射剂[全部字段:智能]) AND (重症肺炎[全部字段:智能]) |
| **Chinese National Knowledge Infrastructure** | TKA=("热毒宁"+"热毒宁注射液"+"热毒宁注射剂")*("重症肺炎") |
| **WanfangData** | 主题:("热毒宁"OR"热毒宁注射液" OR "热毒宁注射剂") AND ("重症肺炎") |
| **VIP** | U=(热毒宁 OR 热毒宁注射液 OR 热毒宁注射剂) AND U=(重症肺炎) |

1. **PRISMA 2020 Checklist**


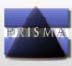
**PRISMA 2020 Checklist**

| **Section and Topic** | **Item #** | **Checklist item** | **Location where item is reported** |
| --- | --- | --- | --- |
| **TITLE** | | |  |
| Title | 1 | Identify the report as a systematic review. | Page1 |
| **ABSTRACT** | | |  |
| Abstract | 2 | See the PRISMA 2020 for Abstracts checklist. | Page4 |
| **INTRODUCTION** | | |  |
| Rationale | 3 | Describe the rationale for the review in the context of existing knowledge. | Page2,4 |
| Objectives | 4 | Provide an explicit statement of the objective(s) or question(s) the review addresses. | Page4 |
| **METHODS** | | |  |
| Eligibility criteria | 5 | Specify the inclusion and exclusion criteria for the review and how studies were grouped for the syntheses. | Page4 |
| Information sources | 6 | Specify all databases, registers, websites, organisations, reference lists and other sources searched or consulted to identify studies. Specify the date when each source was last searched or consulted. | Page4,5 |
| Search strategy | 7 | Present the full search strategies for all databases, registers and websites, including any filters and limits used. | Page4,5 |
| Selection process | 8 | Specify the methods used to decide whether a study met the inclusion criteria of the review, including how many reviewers screened each record and each report retrieved, whether they worked independently, and if applicable, details of automation tools used in the process. | Page5 |
| Data collection process | 9 | Specify the methods used to collect data from reports, including how many reviewers collected data from each report, whether they worked independently, any processes for obtaining or confirming data from study investigators, and if applicable, details of automation tools used in the process. | Page5 |
| Data items | 10a | List and define all outcomes for which data were sought. Specify whether all results that were compatible with each outcome domain in each study were sought (e.g. for all measures, time points, analyses), and if not, the methods used to decide which results to collect. | Page5 |
|  | 10b | List and define all other variables for which data were sought (e.g. participant and intervention characteristics, funding sources). Describe any assumptions made about any missing or unclear information. | Page5 |
| Study risk of bias assessment | 11 | Specify the methods used to assess risk of bias in the included studies, including details of the tool(s) used, how many reviewers assessed each study and whether they worked independently, and if applicable, details of automation tools used in the process. | Page5,6 |
| Effect measures | 12 | Specify for each outcome the effect measure(s) (e.g. risk ratio, mean difference) used in the synthesis or presentation of results. | Page6 |
| Synthesis methods | 13a | Describe the processes used to decide which studies were eligible for each synthesis (e.g. tabulating the study intervention characteristics and comparing against the planned groups for each synthesis (item #5)). | Page8 |
|  | 13b | Describe any methods required to prepare the data for presentation or synthesis, such as handling of missing summary statistics, or data conversions. | None |
|  | 13c | Describe any methods used to tabulate or visually display results of individual studies and syntheses. | Page6,8 |
|  | 13d | Describe any methods used to synthesize results and provide a rationale for the choice(s). If meta-analysis was performed, describe the model(s), method(s) to identify the presence and extent of statistical heterogeneity, and software package(s) used. | Page6,8 |
|  | 13e | Describe any methods used to explore possible causes of heterogeneity among study results (e.g. subgroup analysis, meta-regression). | Page8 |
|  | 13f | Describe any sensitivity analyses conducted to assess robustness of the synthesized results. | Page8 |
| Reporting bias assessment | 14 | Describe any methods used to assess risk of bias due to missing results in a synthesis (arising from reporting biases). | Page8 |
| Certainty assessment | 15 | Describe any methods used to assess certainty (or confidence) in the body of evidence for an outcome. | Page6,8 |
| **RESULTS** | | |  |
| Study selection | 16a | Describe the results of the search and selection process, from the number of records identified in the search to the number of studies included in the review, ideally using a flow diagram. | Page8 |
|  | 16b | Cite studies that might appear to meet the inclusion criteria, but which were excluded, and explain why they were excluded. | Page8 |
| Study characteristics | 17 | Cite each included study and present its characteristics. | Page8,9 |
| Risk of bias in studies | 18 | Present assessments of risk of bias for each included study. | Page9 |
| Results of individual studies | 19 | For all outcomes, present, for each study: (a) summary statistics for each group (where appropriate) and (b) an effect estimate and its precision (e.g. confidence/credible interval), ideally using structured tables or plots. | Page9,10 |
| Results of syntheses | 20a | For each synthesis, briefly summarise the characteristics and risk of bias among contributing studies. | Page9,10 |
|  | 20b | Present results of all statistical syntheses conducted. If meta-analysis was done, present for each the summary estimate and its precision (e.g. confidence/credible interval) and measures of statistical heterogeneity. If comparing groups, describe the direction of the effect. | Page9,10 |
|  | 20c | Present results of all investigations of possible causes of heterogeneity among study results. | Page11 |
|  | 20d | Present results of all sensitivity analyses conducted to assess the robustness of the synthesized results. | Page11 |
| Reporting biases | 21 | Present assessments of risk of bias due to missing results (arising from reporting biases) for each synthesis assessed. | Page11 |
| Certainty of evidence | 22 | Present assessments of certainty (or confidence) in the body of evidence for each outcome assessed. | Page11 |
| **DISCUSSION** | | |  |
| Discussion | 23a | Provide a general interpretation of the results in the context of other evidence. | Page11,12 |
|  | 23b | Discuss any limitations of the evidence included in the review. | Page14 |
|  | 23c | Discuss any limitations of the review processes used. | Page14 |
|  | 23d | Discuss implications of the results for practice, policy, and future research. | Page13,14 |
| **OTHER INFORMATION** | | |  |
| Registration and protocol | 24a | Provide registration information for the review, including register name and registration number, or state that the review was not registered. | Page2 |
|  | 24b | Indicate where the review protocol can be accessed, or state that a protocol was not prepared. | Page4 |
|  | 24c | Describe and explain any amendments to information provided at registration or in the protocol. | None |
| Support | 25 | Describe sources of financial or non-financial support for the review, and the role of the funders or sponsors in the review. | Page14 |
| Competing interests | 26 | Declare any competing interests of review authors. | Page14 |
| Availability of data, code and other materials | 27 | Report which of the following are publicly available and where they can be found: template data collection forms; data extracted from included studies; data used for all analyses; analytic code; any other materials used in the review. | Page14 |

From: Page MJ, McKenzie JE, Bossuyt PM, Boutron I, Hoffmann TC, Mulrow CD, et al. The PRISMA 2020 statement: an updated guideline for reporting systematic reviews. MetaArXiv. 2020, September 14. DOI: 10.31222/osf.io/v7gm2. For more information, visit: <www.prisma-statement.org>

1. **Reduning injection**

**The pathogen of SP can induce a high inflammatory response. RDN injection can address SP for the following reasons: The primary constituents of RDN Injection include *Gardenia jasminoides*, *Lonicera japonica*, and *Artemisia annua*, all of which possess anti-inflammatory, antibacterial, antiviral, and additional properties** ^[1]^. Studies have shown that their active ingredients mainly include iridoids, lignans, coumarins, sesquiterpenes, flavonoids, caffeoylquinic acid and phenolic acids ^[2]^. **The main iridoid in *Gardenia* and chlorogenic acid in *Lonicera japonica* can inhibit the macrophage response associated with inflammatory diseases, reduce the release of inflammatory factors, and inhibit infections** ^[3]^. **A study investigated the antipyretic and anti-inflammatory properties of RDN injection by screening its active ingredients using a mouse endotoxin shock model, resulting in the identification of two novel terpenoid compounds: designated geniposide A and identified as (1*R*,7*R*,8*S*,10*R*)-7,8,11-trihydroxy-4-guaiacin-3-one** ^[4]^. **Research has demonstrated that RDN effectively regulates the metabolic disorders induced by endogenous components in febrile rats treated with dry yeast** ^[5]^. **Its antipyretic effect is mainly related to the regulation of amino acids, lipids, and energy metabolism. Network pharmacology studies have shown that RDN can regulate various biological processes and treat inflammation at the systemic level** ^[6]^. **RDN injection is effective in treating inflammatory diseases, with research indicating that Bcl-2, eNOS, PTGS2, PPARA, and MMPs play a crucial role in modulating the inflammatory processes associated with RDN injection compounds and metabolites** ^[6]^. These mechanisms confirm the authenticity of treating SP with RDN injection.

Each injection of Reduning has a specification of 10 mL and contains 2.6 g of raw drug. The concentration of active ingredients is determined by UPLC-DAD method, and common ingredients include: Chlorogenic acid: approximately 0.12 mg/mL, Chlorogenic acid: approximately 0.29 mg/mL, Geniposide: approximately 0.41 mg/mL, Jingniping gentian glycoside: approximately 0.24 mg/mL, etc;

There have been multiple drug analysis reports on Reduning Injection, involving its component analysis, quality control, fingerprint establishment, and research on allergenic components. In 2025, a quality analysis will be conducted on 45 batches of Reduning Injection according to the national drug standard YBZ0822005-2009-Z, as reported ^[7]^. The results showed that all batches met the requirements and had good quality uniformity, indicating that their production process was stable and suitable for clinical use ^[7]^. In 2021, a study was conducted on the correlation between various active ingredients (such as isochlorogenic acid A, B, caffeic acid, etc.) in Reduning Injection and their antipyretic effects, emphasizing the importance of chemical quality control components in efficacy evaluation ^[8]^. In 2022, a study established an ultra-high performance liquid chromatography-mass spectrometry method for the simultaneous determination of five key components, including geniposide, chlorogenic acid, and chlorogenic acid, in Reduning Injection. The method is accurate, sensitive, and suitable for quality control ^[9]^.

**References:**

1. Xu HM, Wang Y, Liu NF. Safety of an injection with a mixture of extracts from Herba Artemisiae annuae, Fructus Gardeniae and Flos Lonicerae[J]. Pharm World Sci, 2009,31:458-463.
2. Liu J, Sun K, Zheng C, et al. Pathway as a pharmacological target for herbal medicines: an investigation from reduning injection[J]. PLoS One, 2015,10(4):e0123109.
3. Cao YG, Ren YJ, Liu YL, et al. Iridoid glycosides and lignans from the fruits of Gardenia jasminoides Eills[J]. Phytochemistry, 2021,190:112893.
4. Li HB, Yang B, Ge W, et al. Two new terpenoids from Reduning Injection[J]. Chin Herb Med, 2020,12(2):183-187.
5. Gao X, Huang C, Geng T, et al. Serum and urine metabolomics based on UPLC-Q-TOF/MS reveals the antipyretic mechanism of Reduning injection in a rat model[J]. J Ethnopharmacol, 2020,250:112429.
6. Xie F, Xie M, Yang Y, et al. Assessing the Anti-inflammatory Mechanism of Reduning Injection by Network Pharmacology[J]. Biomed Res Int, 2020,2020:6134098.
7. Yang J. Quality Analysis Report of Reduning Injection[J]. Chin Herb Med, 2013,44(24):3493-3495.
8. Fan ZY, Cai LL, Wang YG, et al. Study on the correlation between the antipyretic effect and chemical quality control components of Reduning Injection[J]. Nanjing J Tradit Chin Med, 2021,37(1):126-130.
9. Wang CZ, Nan JL, Dong YL, et al. Simultaneous Determination of Five Components in Reduning Injection by UHPLC-MS/MS[J]. Chin Tradit Pat Med, 2022,44(10):3126-3130.

Table S2 More details about RDN

| Injection name | Source | Species/Raw materials | Botanical plant names | Therapeutic claims in TCM | Indications | Adverse drug reactions | Quality control reported? (Y/N) |
| --- | --- | --- | --- | --- | --- | --- | --- |
| RDN injection | Jiangsu Kanion Pharmaceutical Co., Ltd. | *Artemisiae Annuae Herba,*  *Lonicerae Japonicae Flos,*  *Gardznize Fructus* | *Artemisia annua* L.*,*  *Lonicera japonica* Thunb,  *Gardenia jasminoides* J.Ellis | Clearing heat, dispelling wind, removing toxic substance | Cold due to exogenous wind-heat, marked by high fever, headache, body pain, cough, phlegm yellow; upper respiratory tract infection and acute bronchitis | Dizziness, chest congestion, xerostomia, diarrhea, nausea, vomit, pruritus, skin rash, dyspnea | Y - National Drug Standard YBZ08202005-2009Z and National Pharmaceutical Standard Z20050217 issued by China Food and Drug Administration |
